# Targeting cystatin F activation enhances NK cell cytotoxicity in glioblastoma models

**DOI:** 10.3389/fimmu.2025.1708281

**Published:** 2025-10-28

**Authors:** Emanuela Senjor, Anamarija Habič, Urban Švajger, Ana Mitrović, Matic Proj, Andrej Porčnik, Borut Prestor, Miha Jerala, Matic Bošnjak, Stanislav Gobec, Barbara Breznik, Janko Kos, Milica Perišić Nanut

**Affiliations:** ^1^ Department of Biotechnology, Jožef Stefan Institute, Ljubljana, Slovenia; ^2^ Faculty of Pharmacy, University of Ljubljana, Ljubljana, Slovenia; ^3^ Department of Genetic Toxicology and Cancer Biology, National Institute of Biology, Ljubljana, Slovenia; ^4^ Jožef Stefan International Postgraduate School, Ljubljana, Slovenia; ^5^ Slovenian Institute for Transfusion Medicine, Ljubljana, Slovenia; ^6^ Department of Neurosurgery, University Medical Centre Ljubljana, Ljubljana, Slovenia; ^7^ Institute of Pathology, Faculty of Medicine, University of Ljubljana, Ljubljana, Slovenia; ^8^ Faculty of Chemistry and Chemical Engineering, University of Ljubljana, Ljubljana, Slovenia

**Keywords:** NK cells, cystatin F, glioblastoma, 3D models, microfluidics

## Abstract

**Introduction:**

Glioblastoma (GBM) is a highly invasive brain tumor with limited treatment options and poor prognosis. Natural killer (NK) cells are key effectors of antitumor immunity, capable of eliminating cancer stem-like cells. However, GBM creates an immunosuppressive microenvironment that limits NK cell function. Here, we identify cystatin F as an immunosuppressive factor involved in regulating NK cell granule-mediated cytotoxicity.

**Methods:**

We analyzed cystatin F expression in GBM and its correlation with immune exhaustion markers. NK cell activity was compared between GBM patients and healthy donors. *In vitro* co-cultures of cystatin F-expressing microglial cells and glioblastoma stem-like cells were used to assess NK cell function. To block cystatin F activation from dimeric to active monomeric form, a small-molecule inhibitor of cathepsin V, the activating protease, was applied.

**Results:**

Cystatin F expression correlated with immune exhaustion and suppression markers in GBM. NK cells from patients showed reduced cytotoxicity compared to healthy donors. Co-cultures confirmed that cystatin F-expressing microglia impaired NK cell cytotoxicity, while inhibition of cathepsin V restored NK cell function in standard cytotoxicity assays, 3D spheroids, and microfluidic perfused models.

**Discussion:**

These results indicate that cystatin F mediates NK cell suppression in GBM. Targeting its activation enhances NK cell cytotoxicity, offering a potential strategy to improve NK-based immunotherapy for glioblastoma.

## Graphical Abstract

Increased expression of cystatin F causes decreased cytotoxicity of NK cells, due to its inhibition of granule-mediated cytotoxicity pathway, via the inhibition of cysteine cathepsins C, H and L. For its inhibitory action, cystatin F has to be converted to monomeric form, which is facilitated by cathepsin V. By inhibiting cathepsin V with compound 7, we can mitigate immunosuppressive effects of cystatin F on NK cell cytotoxicity.

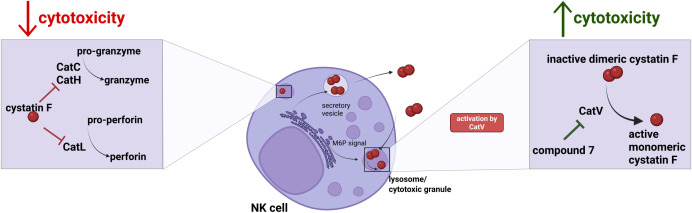

## 1 Introduction

Natural killer (NK) cells have an important role in antitumor immune response as they can eliminate cancer cells, especially cancer stem cells, without prior antigen sensitization (1). While NK cells have several mechanisms for targeting tumor cells, the main mechanism against tumor cells is granule-mediated cytotoxicity. The release of these granules, which contain granzymes and perforin, into the immune synapses causes a cascade of reactions that lead to the apoptosis of target cells (2). Cysteine cathepsins regulate the activity of the cytotoxic molecules inside these granules. More specifically, cathepsins C and H are important for granzyme activation, while perforin is converted to the active form by cathepsin L (3, 4). The functionality of NK cells in cancer is often compromised. Due to the reduced cytotoxic activity and cytokine secretion capacity, NK cells in cancer patients are less effective in eliminating tumor cells. Several immunosuppressive factors contribute to the reduced functionality of NK cells (5–13), among them cystatin F, an endogenous inhibitor of cysteine peptidases. Cystatin F is a N-glycosylated protein. The glycosylation enables either secretion and internalization into bystander cells, or translocation to lysosomes and cytotoxic granules (14–17). The latter is a place where cystatin F is converted from the dimeric form, which is inactive, to the monomeric form, which can inhibit cathepsins C, H, and L. This conversion is catalyzed by cathepsin V (18, 19). Cathepsin V is one of the lesser-known cathepsins (20). Its expression is elevated in several cancers such as bladder (21), breast (22–24), liver (25–27), kidney (28), lung (29, 30), gastric (31), and uterine malignancies (32). It is associated with the processes of cell proliferation (27), as its expression correlates with cell cycle and regulatory genes (23), or activation of NF-κB signaling (21). It might also be involved in promoting metastasis by downregulating adhesion molecules (22, 30). Its expression is also linked to the infiltration of tumor-associated macrophages (TAMs) in hepatocellular carcinoma (33) and lung cancer (30), moreover, it is involved in TME remodeling by exhibiting elastolytic activity in activated macrophages (34). In this study, we mainly focus on the characteristic that cathepsin V activates cystatin F to its monomeric form, and therefore contributes to the reduced cytotoxic potential of NK cells (18, 19). Cystatin F’s expression is elevated in several types of cancer, such as colorectal cancer, liver metastasis, pancreatic ductal adenocarcinoma (35–37), and glioblastoma (GBM), as shown in our previous study (16). Increased cystatin F levels are correlated with poor prognosis in glioblastoma (16) and contribute to immunosuppression by reducing the cytotoxicity of immune cells (16, 38–40). There is a lot of inter- and intra-tumor heterogeneity in GBM, where, in addition to dynamic cell states of tumor cells, non-tumor cells, such as immune and endothelial cells, are present (41). Additionally, the tumor microenvironment in glioblastoma is populated with therapy-resistant GBM stem-like cells (GSCs) (42). The GBM tumor microenvironment is often described as immunosuppressive, which also limits the efficiency of immunotherapeutic approaches (43). Complex and dynamic interactions within the TME drive therapeutic resistance, which prevents efficient treatment. The interplay between immune cells and glioblastoma cells is crucial for understanding both the immune evasion mechanisms of the tumor and potential therapeutic strategies. To develop new approaches for the treatment of glioblastoma, appropriate tumor models are of the essence. While standard 2D models offer better reproducibility and ease of use, they usually fail to adequately mimic the complexity of tumors. Using advanced models such as 3D spheroids or ex vivo cultures in static or perfused conditions using microfluidic devices brings us closer to the complexity of the tumor environment *in vivo* (44). The aim of this study was to evaluate the inhibition of cystatin F activation in 2D and simple 3D models using GBM GSCs, with the goal of improving the cytotoxic potential of NK cells.

## 2 Materials and methods

### 2.1 TIMER database analysis

Gene-level correlations with *CST7* expression and tumor purity and other TME-related genes were performed using publicly available data of Pearson correlation coefficients and associated p-values between CST7, tumor purity and other genes from TIMER database (accessed in March 2025). Data was processed in R using tidyverse and pheatmap packages. Full correlations (between *CST7* or tumor purity and all other genes) and partial correlations adjusted for tumor purity between *CST7* and other genes were extracted. A clustered heatmap was generated to visualize correlations. Clusters were defined by cutting the dendrogram into three groups, based on the correlations with *CST7*. Scatter plots were generated to compare full and purity adjusted correlations of each gene with *CST7*, points are colored based on a gradient of purity-adjusted correlation values. The dashed line at 45° angle shows the differences in full and adjusted correlations (points lying directly on this line would indicate that full and partial correlations are identical, whereas for points below the dashed line the correlation is reduced after adjustment).

### 2.2 Human samples: tissue and PBMCs

GBM tissue and PBMC samples were obtained from the GlioBank established by the National Institute of Biology Slovenia. Resected tumor tissue is obtained by the Department of Neurosurgery, University Medical Centre Ljubljana, Slovenia (45). The study was approved by the National Medical Ethics Committee of the Republic of Slovenia (Approval no. 0120-190/2018-2711-41).

Buffy coats of healthy volunteers were obtained and processed at the Slovenian Institute for Transfusion Medicine according to institutional guidelines (approval no. 0120-279/2017-3).

### 2.3 Immunohistochemistry

Brain samples of 2 patients with newly diagnosed GBM were included in this study. Paraffin-embedded tissue sections (5 μm) were prepared according to standard procedures at the Institute of Pathology, Medical Faculty, University of Ljubljana, Slovenia. The sections were then deparaffinized in 100% xylene (2 × 3 min) and rehydrated in 100%, 96% and 70% ethanol and, finally, dH2O for 3 min each. Heat-induced antigen retrieval was performed for 20 min in citrate buffer (pH = 6.0). Non-specific background binding was reduced by incubating the tissue sections in 10% horse serum (Sigma, USA) in phosphate-buffered saline (PBS) containing 1% bovine serum albumin (BSA) and 0.1% Triton-X. Subsequently, the serum was removed and the sections were incubated overnight at 4 °C with the following primary antibodies: rabbit anti-cystatin F (1:200, Sigma, HPA040442, USA), mouse anti-CD45 (1:100, Invitrogen, 14-9457-82), mouse anti-LAG3 (1:100, R&D MAB 231971), mouse anti-TIM3 (1:100, Invitrogen, MA5-32841) mouse anti-PD1 (1:100, R&D, MAB1086) and mouse anti-DNAM1 (1:100, R&D, MAB6661). Next, the sections were incubated in secondary goat anti-rabbit Alexa Fluor 488 (A11008) and donkey anti-mouse Alexa Fluor 546 (A10036) antibodies or donkey anti-mouse Alexa Fluor 647 (A21236), Thermo Fisher Scientific, USA, for 1 h (1:200), protected from light. Nuclei were stained with Hoescht 33258 solution (1:1000) and cover-slipped with ProLong Gold antifade mountant (Invitrogen, P36980). Confocal microscopy images were obtained with confocal microscope (LSM 710, Carl Zeiss, Germany) and ZEN 2.3 SP1 FP1 software (Carl Zeiss, Germany).

### 2.4 Cell lines

K-562 cells were cultured in RPMI 1640 (Gibco, Massachusetts, USA), 10% heat-inactivated fetal bovine serum (HI-FBS) (Gibco) and 1% penicillin/streptomycin (P/S) (Gibco). HMC3 cells were cultured in EMEM (ATCC) and 10% heat-inactivated fetal bovine serum (HI-FBS). NCH-421k were obtained from CLS (Cell Lines Service GmbH, Eppelheim, Germany) and grown as spheroid suspensions in complete Neurobasal Medium (Invitrogen, Life Technologies, Carlsbad, CA, USA) containing 2 mM L-glutamine, 1 × penicillin/streptomycin, 1 × B-27 (Invitrogen), 1 U/mL heparin (Stem Cell technologies, Vancouver, Canada), 20 ng/mL β-FGF and EGF (both Invitrogen). K-562 and HMC3 cell lines were obtained from ATCC. All cell lines tested negative for mycoplasma contamination and were cultured at 37 °C and 5% CO2.

### 2.5 NK cell isolation

Peripheral blood mononuclear cells were collected after Ficoll-assisted gradient centrifugation at the Blood Transfusion Centre of Slovenia. NK cells were isolated using a magnetic NK cell isolation kit with negative selection (cat. 130-092-657, Miltenyi Biotec, Germany). The purity of the isolated cells was checked with a flow cytometer (Attune NxT, Thermo Fisher Scientific, Massachusetts, USA) with anti-CD3 (cat. 130-113-128, Miltenyi Biotec), anti-CD56 (cat. 47056742, Invitrogen), anti-CD16 (cat. 46016642, Invitrogen) antibodies. FlowJo software (BD Life Sciences, Oregon, USA) was used for analysis.

Primary NK cells were cultured in RPMI 1640 media (Gibco) supplemented with 8% HI-FBS, MEM non-essential amino acids (Gibco), sodium pyruvate (Gibco), 1% P/S, and 1000 IU/mL IL-2 (cat. 4030758, Bachem, Switzerland).

### 2.6 Western blot

Post-nuclear cell lysates were prepared in RIPA buffer containing protease inhibitors (Roche) and obtained after 30 min incubation on ice and 30 min centrifugation at 16 000g. Total protein concentration was measured using the DC protein assay (BioRad, California, USA). Non-reducing SDS PAGE was performed, and proteins were transferred to nitrocellulose membranes using the Trans-Blot Turbo system (BioRad). Membranes were blocked in 5% non-fat dry milk in PBS for 1 h and incubated overnight with primary antibodies. After incubation with HRP-conjugated secondary antibodies, the Clarity Max ECL substrate (BioRad) was used to visualize the bands in the ChemiDoc MP imaging system (BioRad). Blots were analyzed using Image Lab software (BioRad).

The following antibodies were used: rabbit anti-cystatin F (cat. HPA040442, Sigma), rabbit anti-cathepsin V (cat. ab166894 abcam, Cambridge, UK) or goat anti-cathepsin V (cat. AF1080, R&D) primary antibodies, and anti-rabbit/sheep HRP (cat. 111-035-045, 313-035-003, Jackson Immuno Research, Pennsylvania, USA), and anti-mouse StarBright 700 secondary antibodies (cat. 12004158, BioRad). Signal normalization analyses were performed using Stain-Free technology (BioRad).

### 2.7 Immunocytochemistry

Coverslips were coated with poly-L-lysine (Sigma). After cells adhered to the coverslips, they were fixed with 4% paraformaldehyde/PBS for 20 min, washed with PBS, and permeabilized with 0.1% Triton X-100 for 10 min. The coverslips were then blocked with 3% BSA/PBS for 1 h at room temperature. Cells were then labelled with rabbit anti-cathepsin V (cat. ab166894 abcam) primary antibodies for 1 h at room temperature. After washing, coverslips were incubated with the secondary antibodies anti-rabbit 488 (cat. 4412S, Cell Signaling Technology) for 1 h at room temperature. Cells were then washed and stained with DAPI (Sigma-Aldrich) and mounted on slides overnight with ProLong Gold Antifade reagent (P36930, Thermo Scientific). Slides were analyzed using a LSM 710 confocal microscope (Carl Zeiss) and ZEN 2.3 SP1 FP1 software.

### 2.8 NK cell cytotoxicity

#### 2.8.1 Calcein-AM release assay

NK cells were activated overnight with 1000 IU/mL IL-2 and prepared at selected effector-to-target ratios. K-562 target cells were labelled with 15 µM calcein-AM (cat. 17783, Sigma) in serum-free media for 30 min. The cells were washed and added (n = 5000) to effector cells. The plate was centrifuged at 200g for 1 min and incubated for 4 h at 37 °C with 5% CO2. After incubation, the plate was centrifuged at 700g for 5 min, and 50 μL of supernatant was transferred to a new microtiter plate for fluorescence measurements. Released calcein-AM was measured using the Tecan M1000 microplate reader at 496 nm excitation and 516 nm emission. The percentage of cytotoxicity was calculated as: 100 × (test release − spontaneous release)/(total release − spontaneous release). Spontaneous release of calcein-AM was measured in wells containing 100 μL of NK culture media and 50 μL of target cells. For total release, 2% Triton X-100 was added to the NK culture media to achieve target cell lysis. LU30 were calculated using the inverse of the number of effector cells needed to lyse 30% of the target cells, normalized to 10^6^ effector cells.

#### 2.8.2 Flow cytometry cytotoxicity assay

NK cells were activated overnight with 1000 IU IL2/ml and treated with 20 µM Compound 7, established in our previous study (19). NK cells were then prepared in selected effector-to-target ratios, with 5–8 serial dilutions in 96-well U-bottom plates. Meanwhile, target NCH-421k cells were stained with 0.2 µM carboxyfluorescein succinimidyl ester (CFSE) (cat. C34554, Invitrogen) for 20 min at 37 °C, washed with media, and added (n = 20 000) to effector cells. The plate was centrifuged at 200g for 1 min and incubated for 4 h at 37 °C. Each well was transferred to a flow tube. The cells were washed twice and resuspended in PBS. 7-Amino-actinomycin D (7-AAD) was added to each flow tube (cat. SML1633, Sigma). After incubation on ice for 10 min, the samples were run in an Attune NxT flow cytometer (Thermo Fisher Scientific). The results were analyzed with FlowJo. Briefly, spontaneous lysis of target cells (wells that contained only target cells but no effector cells) was subtracted from the percentage of dead target cells (CFSE and 7-AAD positive cells) determined for each sample. Lytic units (LU) 30/10^6^ cells were calculated using the inverse of the number of effector cells needed to lyse 30% of the target cells, normalized to 10^6^ effector cells.

For evaluation of cytotoxicity of NK cells against NCH-421k and HMC3 cocultures, NCH-421k cells were stained with CFSE as described above. HMC3 cells (control and CSTF WT transfected) were stained with CellTracker Deep Red (cat. C34565, Invitrogen). After staining, cells were mixed in 2:1 ratio (NCH-421k: HMC3). NK cells were activated overnight with 1000 IU IL2/mL, and then prepared at selected effector-target ratios and plated in 96-well U-bottom plates together with target cells, as described above. After the incubation and wash steps, Helix NP Blue dead dye (cat. 425305, BioLegend, California, USA) was added to each sample and measured using Attune NxT flow cytometer (Thermo Fisher Scientific). The results were analyzed with FlowJo. Briefly, spontaneous lysis of target cells (wells that contained only target cells but no effector cells) was subtracted from the percentage of dead target cells for NCH-421k (CFSE and Helix NP positive cells) and HMC3 cells (CellTracker Deep Red and Helix NP positive cells) determined for each sample.

### 2.9 Confocal microscopy NK cytotoxicity - static conditions

CFSE-labeled NCH-421k spheroids were placed in imaging 96-well plates (Greiner Bio One, 655976). Then, NK cells (which were activated with 1000 IU IL2/mL and treated overnight with 20 µM compound 7) were added to the wells in 5:1 ratio, together with 200 IU/mL of IL2 and 20 µM of compound 7 or 0.1% DMSO and incubated for 3 days. Z-stacks of the spheroids were imaged using confocal microscope LSM710 (Carl Zeiss). ImageJ software with StarDist (46), TrackMate (47) and 3Dsuite (48) plugins was used for quantitative analysis. Briefly, each z-stack was segmented using StarDist plugin to segment individual cells based on the CFSE signal. Obtained ROIs were imported to 3DSuite 3D manager, where the CFSE intensity of each individual ROI was measured. Then we calculated the mean intensity of all ROIs in individual spheroid and used this value to calculate the percentage of dead cells as a decrease in the CFSE signal intensity in spheroids treated with pNK (with or without inhibitor) compared to control spheroids that were not treated with pNK, using this equation:


$$\%dead\;cells=100\times \frac{meanCFSE\;of\;ctrl\;spheroid-mean\;CFSE\;of\;spheroid+NK}{meanCFSE\;of\;ctrl\;spheroid}$$

### 2.10 Confocal microscopy - perfused conditions

NCH-421k cells were labeled with 5 µM CellTrace Violet (Invitrogen, C34557) for 15 minutes at 37 °C. After washing, 25–000 cells were loaded in the IdenTx microfluidic chip (AIM Biotech, Singapore) in complete NBE media with or without the addition of 10% matrigel (Corning). Side compartments were loaded with NBE complete media according to manufacturer’s instructions and incubated overnight. NK cells were activated with 1000 IU IL2/mL and treated overnight with 20 µM compound. Next day, NK cells were labeled with 2 µM CellTracker Deep Red for 15 minutes at 37 °C. After washing, NK cells were loaded in one of the media channels, in 5:1 ratio, with the addition of 200 IU/mL of IL2, 500 nM Cytotox Green dye (Sartorius) and 20 µM compound 7 or 0.1% DMSO. The media inlets were filled with a difference in volume in order to generate interstitial flow across the chip, according to manufacturer’s instructions. Chips were incubated overnight and then imaged using confocal microscopy (LSM 710, Carl Zeiss) or widefield microscopy (EVOS M5000 Imaging system, Thermo Fisher). Images were taken on the whole length of the chip. Images were analyzed using ImageJ software. StarDist plugin (46) was used to segment tumor cells. Then the area of segmented ROIs was measured. Intensity of the cytotoxicity dye was measured inside the segmented ROIs. Intensity of the dead dye for each field of view was normalized to the segmented tumor cell area. Values from the NCH-421k DMSO-treated cells without the addition of NK cells were used for normalization. Since different microscopes were used for image acquisition, we show the results of each individual experiment.

### 2.11 ELISA

The ELISA Max Standard Set Human IFNγ kit (cat. 430104, BioLegend) was used to measure IFNγ in NK cell supernatants, according to manufacturer’s instructions. Absorbance at 450 nm was measured with a microplate reader (Infinite M1000, Tecan, Switzerland).

### 2.12 Multiplex ELISA

Luminex assay was performed in supernatants from NK cells isolated from healthy donors or GBM patients that were activated overnight with 1000 IU/mL IL-2 (Bachem). R&D Luminex Human Immunotherapy 25-plex Fixed panel was used (LKTM010B, Biotechne, Minneapolis, Minnesota, USA) and measured with Luminex platform (MAGPIX System, Merck, Darmstadt, Germany) according to manufacturer instructions.

### 2.13 Cell transfection and HMC3

HMC3 cell line transfection was performed using cystatin F WT pcDNA3 plasmid prepared as described in (38) using lipofectamine 3000 transfection reagent (Invitrogen, Life Technologies, Carlsbad, CA, USA), according to manufacturer instructions. Control HMC3 cells were prepared similarly, without the addition of the plasmid. After three days, the cells were used in NK cytotoxicity experiment.

### 2.14 Spheroid formation and staining

Hanging drop method was used to form spheroids from NCH-421k cells. Cells were first stained with CFSE (5 µM) for 15 min at 37 °C. After washing step, cells were resuspended in complete neurobasal medium containing 4% methylcellulose/NBE media mixture. 4% methylcellulose was prepared by autoclaving 6 g of pure methylcellulose powder (Sigma, catalog # m-0512), dissolving it in 250 ml pre-heated complete Neurobasal medium (60 °C) for 20 min using a magnetic stirrer. Then 250 ml room temperature complete Neurobasal medium was added, to a final volume of 500 ml. This solution was mixed overnight at 4 °C. The final stock solution was aliquoted and cleared by centrifugation (5000×*g*, 2 h, room temperature). 20 µL drops were formed on the lid of 10 cm^2^ plate, containing 5000 cells. The lid was then inverted over dishes containing PBS to prevent evaporation. After 3 days, the spheroids were ready for further experiments.

### 2.15 Tumor cell proliferation

#### 2.15.1 Flow cytometry proliferation assay

NCH-421k cells were labeled with CFSE as described above. Cells were then washed and resuspended in NBE complete media and treated with 20 µM compound 7 of 0.1% DMSO for 48h. Cells were then collected into round-bottomed polystyrene tubes and washed with PBS. Before measurement, Helix NP dye (BioLegend) was added to exclude dead cells. CFSE signal was measured using Attune NxT flow cytometer (Thermo Fisher), and analyzed with FlowJo.

#### 2.15.2 Spheroid proliferation assay – static condition

NCH-421k spheroids were prepared using hanging drop method, as described above. Spheroids were then transferred to imaging 96- well plates and treated with 20 µM compound 7 of 0.1% DMSO for 3 days. The spheroids were imaged on day 0 and on day 3 with EVOS 5000 Imaging system (Thermo Fisher). Brightfield images were taken in a way, that the outer border of spheroids was in focus, in order to reproducibly image the largest spheroid area. Spheroid area was measured using AnaSP software (49). Area of spheroids on day 3 was normalized to the area at day 0.

#### 2.15.3 Spheroid proliferation assay – perfused condition

For experiments evaluating spheroid proliferation in perfused conditions, NCH-421k cells were not stained prior to hanging drop formation. The drops contained 1000 cells to accommodate the size of the cell compartment in the microfluidic chip. Fluigent (France) LineUp series was used for the microfluidic setup. Briefly, the setup was composed of a pressure source, 2 FlowEZ pressure controllers, SwitchEZ, which controls the Flow units, which enable the flow rate regulation inside the system. Media was stored inside a thermoblock, ensuring perfusion with media heated to 37 °C. The system was controlled via the OxyGEN software (Fluigent). Before each experiment, the tubing and the microfluidic setup was sanitized with ethanol. After sanitization, the system was primed with NBE media. µ-Slide Spheroid Perfusion chip (Ibidi, Germany) was used for the evaluation of spheroid growth in perfused conditions. The slides were incubated at 37 °C overnight to release any gases absorbed in the plastic. Individual spheroids were transferred into the wells. The chip was then sealed with the coverslip, and NBE complete media was added to hydrate the media channels. Chip was then incubated at 37 °C for 2h for equilibration. Using Luer adapters, the Ibidi chip was connected to the Fluigent microfluidic setup. Flow rate was set to 0.5 µL/min (or 30 µL/h). Spheroids were imaged using EVOS M5000 imaging system at day 0 and after 3 days of perfusion. The area of the spheroids was measured using AnaSP software as described above. Area of spheroids on day 3 was normalized to the area on day 0.

### 2.16 Statistics

GraphPad prism 10 software was used to perform statistical analysis (t-test or ANOVA as required). Values are presented as mean ± SD, and statistical significance was set at p< 0.05.

## 3 Results

### 3.1 NK cells have impaired function in glioblastoma patients

**Figure 1:**
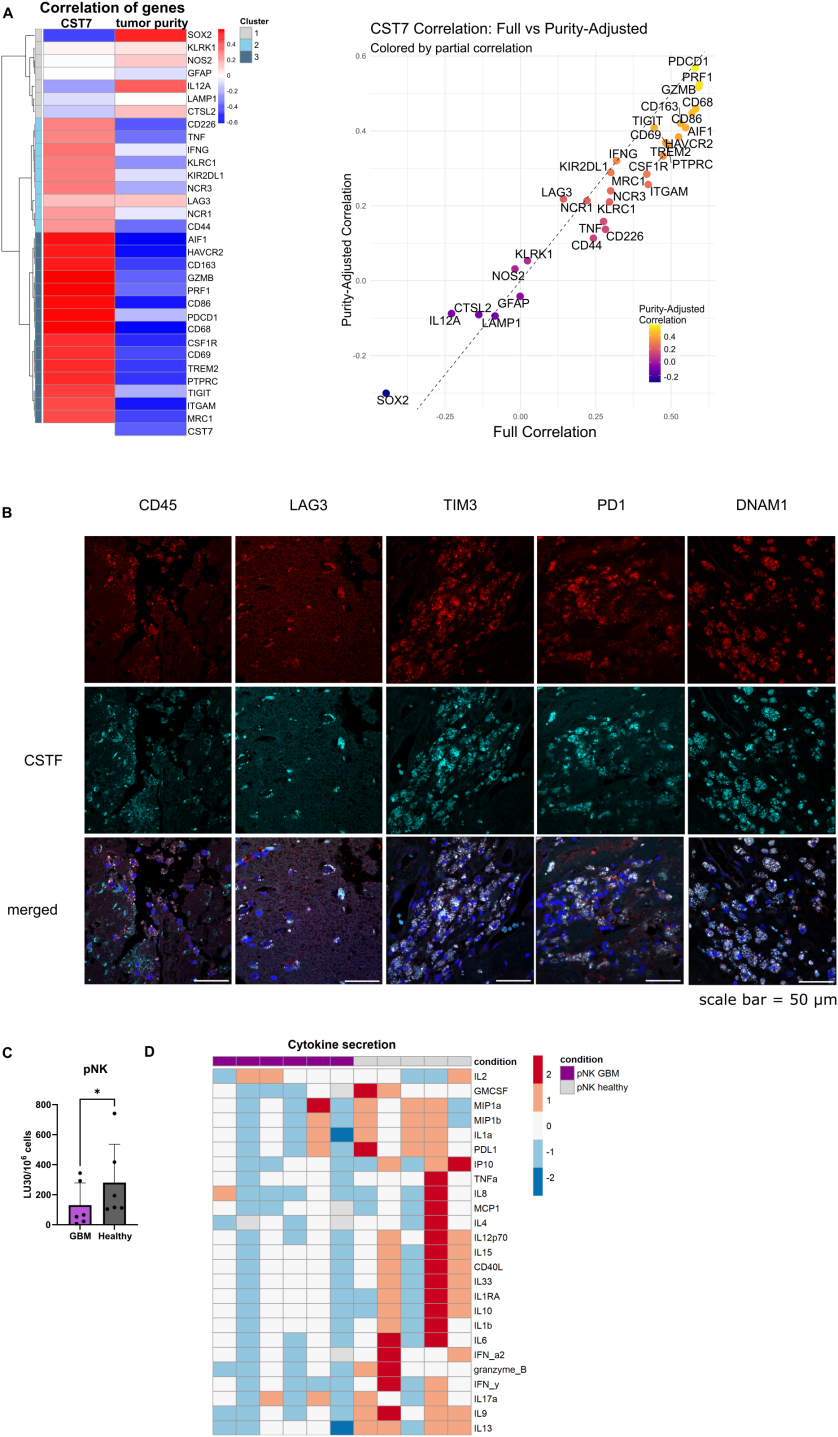
NK cells from GBM patients have reduced functionality. **(A)** Left: Heatmap for gene correlations of various tumor related genes (*SOX2, GFAP, CTSL2*), general immune-related genes (*PTPRC* (CD45)), monocyte/microglia related genes (*CD68*, *AIF1* (Iba-1), *MRC1, TREM2, CSF1R, CD163, NOS2*), immune activation (*CD226* (DNAM1), *NCR1* (NKp46), *NCR3* (NKp30), *KLRK1*(NKG2D), *CD69, CD86, IL12A, LAMP1, TNF, IFNG, GZMB, PRF1*) and immune inhibition related genes (*KIR2DL1, KLRC1* (NKG2A), *PDCD1* (PD1), *TIGIT, HAVCR2* (TIM3), *LAG3*) and immune-adhesion related genes (*ITGAM* (CD11b), *CD44*) with cystatin F (*CST7*) and tumor purity. Red indicates positive correlation; blue indicates negative correlation. Right: Scatter plot comparing full correlation of each gene with *CST7* against the purity-adjusted correlation with *CST7*. Genes above the dashed line show increased correlation after purity adjustment and those below show decreased correlation. Data was obtained from TIMER database, accessed in March 2025. **(B)** Co-expression of cystatin F with various immune markers in GBM tissue sections from two patients. Scale bar= 50 µm. **(C)** Calcein-AM release cytotoxicity assay of primary NK cells from healthy donors (n=6) or GBM patients against (n=6) K-562 target cells. LU 30/10^6^ cells were calculated using the inverse of the number of effectors needed to lyse 30% of the tumor cells. **(D)** Z-score representation of cytokine secretion from healthy donors’ NK cells (n=5) or GBM patients’ NK cells (n=6) using multiplex ELISA. *p<0.05.

Publicly available data from TIMER database reveals a correlation of cystatin F gene (*CST7*) expression in glioblastoma with immune infiltration (negative correlation with tumor purity) (**Figure 1A**, left). Selected genes, relevant to cytotoxic immune cells, microglia/macrophages, and tumor-associated genes, were clustered based on the correlation strength with *CST7* into three clusters. Tumor-associated genes and some immune activation genes in cluster 1 had a weak correlation with *CST7*. Genes with moderate correlation with *CST7* connected to the functional immune responses (NK cell activation, inhibitory receptors, and cytokine genes) are in cluster 2. Genes in cluster 3, associated with microglia/macrophages, cytotoxic immune cell activation, and immunosuppression signatures (M2 markers or immune checkpoints), had the highest correlation with *CST7* expression (**Figure 1A**, left). Additionally, the full correlations and purity-adjusted correlations were plotted in order to consider the effect of tumor composition on the correlations between immune-related genes (**Figure 1A**, right). Detailed information from the TIMER database can be found in **Supplementary Figure S1A**. We have confirmed co-expression of cystatin F with immune-related markers (CD45, LAG3, TIM3, PD1 and DNAM1) on a protein level in glioblastoma tissue sections (**Figure 1B**). Next, we have analyzed the function of NK cells isolated from glioblastoma patients and compared it to the function of healthy donors’ NK cells. As it was shown for other cancer types (5, 6, 11, 12), NK cells from glioblastoma patients have reduced cytotoxic function against standard target cells K-562 (**Figure 1C**). %-cytotoxicity plots are shown in **Supplementary Figure S1B**. Furthermore, GBM patients’ NK cells also secrete a low amount of various cytokines, as analyzed by multiplex ELISA (**Figure 1D**; **Supplementary Figure S1D**). Patients’ NK cells also expressed higher levels of cystatin F compared to healthy donors (**Supplementary Figure S1C**). From this data, we can conclude that GBM patients’ NK cells have reduced functionality compared to healthy donors.

### 3.2 Cystatin F from the tumor microenvironment negatively affects NK cell cytotoxicity

**Figure 2:**
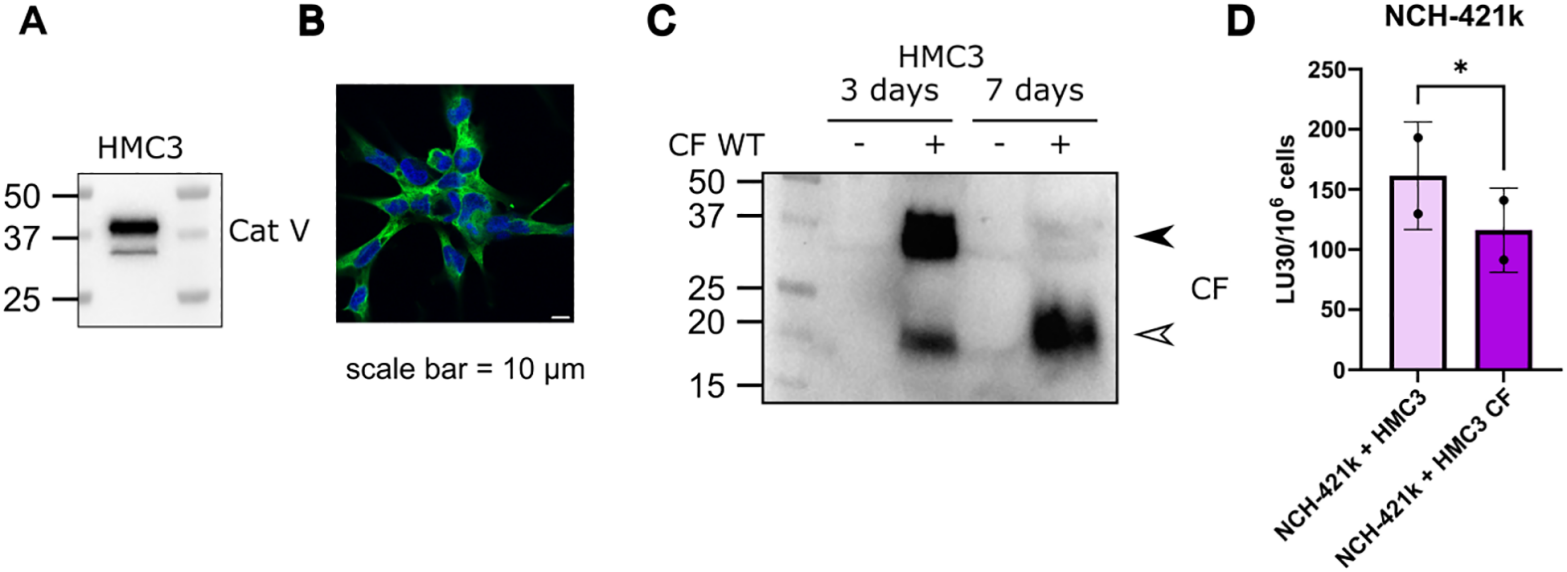
Cystatin F presence in the TME can negatively impact NK cell cytotoxicity. **(A)** Microglia HMC3 cell line expresses cathepsin V, confirmed by western blot and **(B)** immunocytochemistry. Scale bar 10 μm. **(C)** Microglia cell line HMC3 transfected with CF WT, retains expression of cystatin F, and due to the presence of cathepsin V, cystatin F gets activated to monomeric form. Arrowheads point to the dimeric form (black) and monomeric form (white) (left). **(D)** In co-culture NK cytotoxicity experiments with NCH-421k and HMC3 cells the presence of cystatin F decreases NK cell cytotoxicity against NCH-421k cells (n=2). *p<0.05.

We have previously shown deleterious effects of cystatin F on NK cell function, using either recombinant cystatin F or conditioned media containing cystatin F (16, 38). Here we show that the presence of microglial cells expressing cystatin F can negatively affect NK cell cytotoxicity. We have used the HMC3 microglial cell line (50), that does not express cystatin F on a protein level, however, it expresses cathepsin V, which is crucial for the activation of cystatin F from its dimer to monomer form (**Figures 2A, B**). We have transfected the HMC3 cell line with cystatin F and confirmed its expression and activation to the monomeric form even 7 days after transfection (**Figure 2C**). This conversion was limited when HMC3 cells were treated with the cathepsin V inhibitor, compound 7 (19) (**Supplementary Figure S2D**). Furthermore, transfected HMC3 cells also secreted cystatin F into the culture media (**Supplementary Figure S2E**). Next, we have performed flow cytometry cytotoxicity assay, where we have prepared co-cultures of glioblastoma stem cells NCH-421k (labeled with cell trace violet), with the control or transfected HMC3 cells (labeled with cell tracker deep red). NK cells from healthy donors were less effective in eliminating NCH-421k cells in the presence of the transfected HMC3 cell line (**Figure 2D**). %-cytotoxicity plots are shown in **Supplementary Figure S2F**. HMC3 cells are not targeted by NK cells (**Supplementary Figure S2G**).

### 3.3 Negative effects of cystatin F on NK cell cytotoxicity can be reduced by preventing its activation to the monomeric form

**Figure 3:**
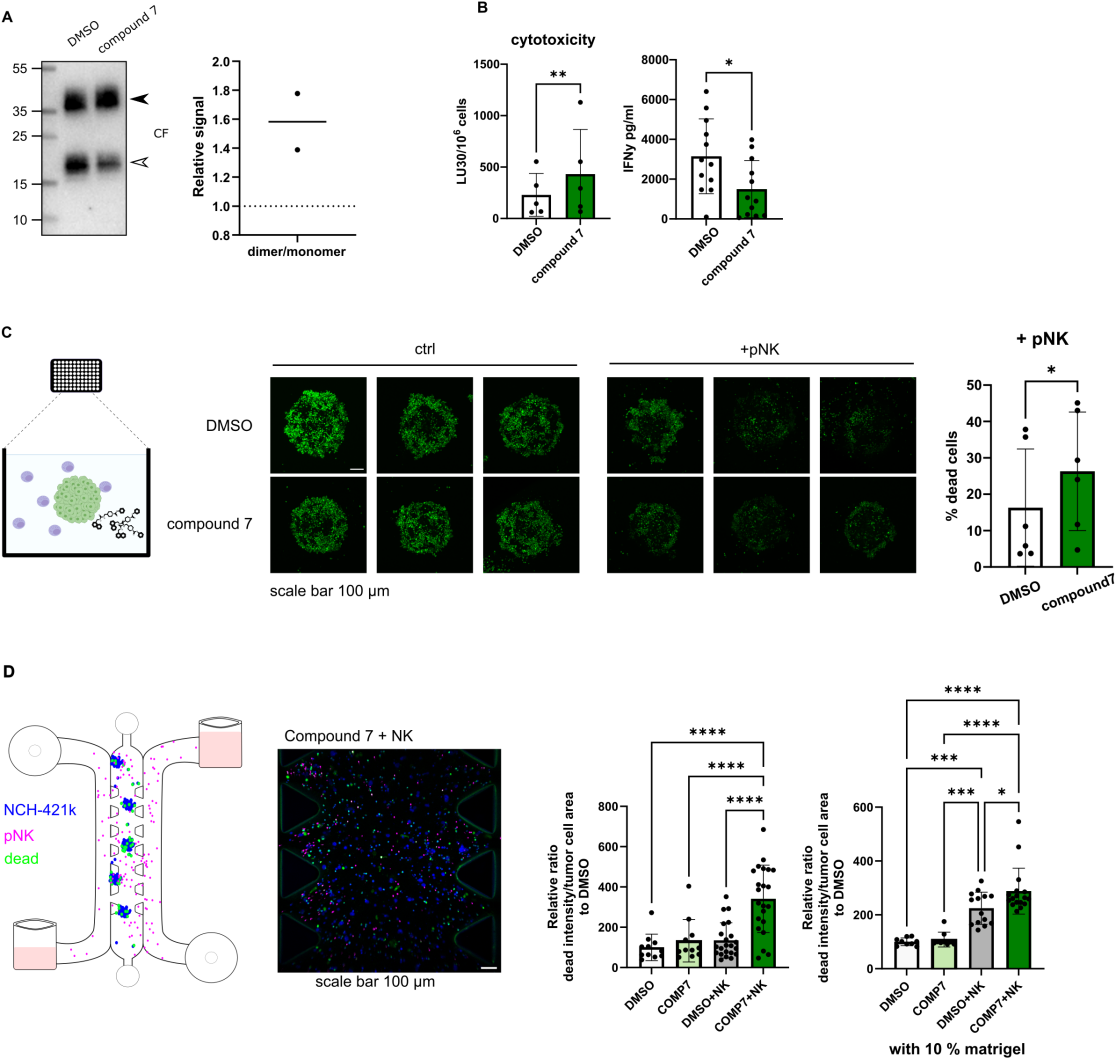
Cytotoxic potential of NK cells can be improved by preventing the activation of cystatin F. **(A)** Representative western blot of cystatin F in primary NK cells (pNK) treated with DMSO or compound 7. Arrowheads point to the dimeric form (black) and monomeric form (white) (left). Quantification of cystatin F dimer/monomer ratio (n=2) (right). Stain free loading control is shown in **Supplementary Figure S3A**. **(B)** Treatment of pNK cells with compound 7 (20 µM, 18h) improves pNK cell cytotoxicity against single cell suspension NCH-421k cells (CFSE/7-AAD assay) (left) (n=5). IFNγ secretion in control (DMSO) or compound 7 treated pNK cells measured by ELISA (right) (n=12). **(C)** Treatment of pNK cells with compound 7 (20 µM, 18h) improves pNK cell cytotoxicity against NCH-421k spheroids cultured in static conditions. Schematic representation of the experiment (left). Representative max projections of z-stacks, scale bar 100 μm (middle). Quantification of dead cells in spheroids treated with pNK cells and DMSO or compound 7 for 3 days (n=6). % of dead cancer cells was determined as the decrease in CFSE signal in NK-treated spheroids compared to the control spheroids, which were not exposed to NK cells (right). **(D)** Treatment of pNK cells with compound 7 (20 µM, 18h) improves pNK cell cytotoxicity against NCH-421k cells cultured in a microfluidic device. Schematic representation of the experiment (left). Representative image of loaded microfluidic device, scale bar 100 µm. (middle). Quantification of dead cancer cells after 1 day. Cancer cells were labeled with CellTrace Violet (blue), pNK cells were labeled with CellTracker Deep Red (magenta), and dead cells were labeled with Cytotox Green (green). The intensity of Cytotox Green signal within segmented tumor areas was normalized to tumor cell area. DMSO only condition was used for normalization. The whole length of the microfluidic device was imaged and used for quantification. Individual experiments are represented for culture conditions without extracellular matrix and the addition of 10% matrigel to the microfluidic device (right). Each dot represents a field of view. *p<0.05, **p<0.01, ***p<0.001, ****p<0.0001.

In our previous study, we developed compound 7 (structure shown in **Supplementary Figure S3E**), a selective, non-covalent and reversible inhibitor of cathepsin V, a peptidase converting dimeric cystatin F to monomeric active form (19). Here we show that compound 7 is effective in reducing cystatin F activation in primary NK cells derived from healthy donors (**Figure 3A**). Reduced levels of active cystatin F are reflected in increased cytotoxic potential of NK cells, as they were more effective in eliminating GBM stem cell line NCH-421k, as shown by flow cytometry cytotoxicity assay (**Figure 3B**). %-cytotoxicity plots are shown in **Supplementary Figure S3C**. Treatment of NK cells with compound 7 also decreased the secretion of IFNγ (**Figure 3B**). In the next step, we prepared tumor spheroids from NCH-421k cells and exposed them to NK cells from healthy donors and cathepsin V inhibitor under static conditions (**Figure 3C**). Spheroids were imaged using confocal microscopy after 3 days. NK cells were more successful in eliminating tumor cells in the spheroids in the presence of compound 7 (**Figure 3C**). Lastly, we have performed a similar experiment using microfluidic chip device, where we have introduced perfusion. Due to difference in level of added media in the media channel, interstitial flow was formed in the chip (51). Like in the static model, NK cells were more effective in eliminating tumor cells in the presence of compound 7 (**Figure 3D**).

### 3.4 Cathepsin V inhibitor affects tumor cell proliferation

**Figure 4:**
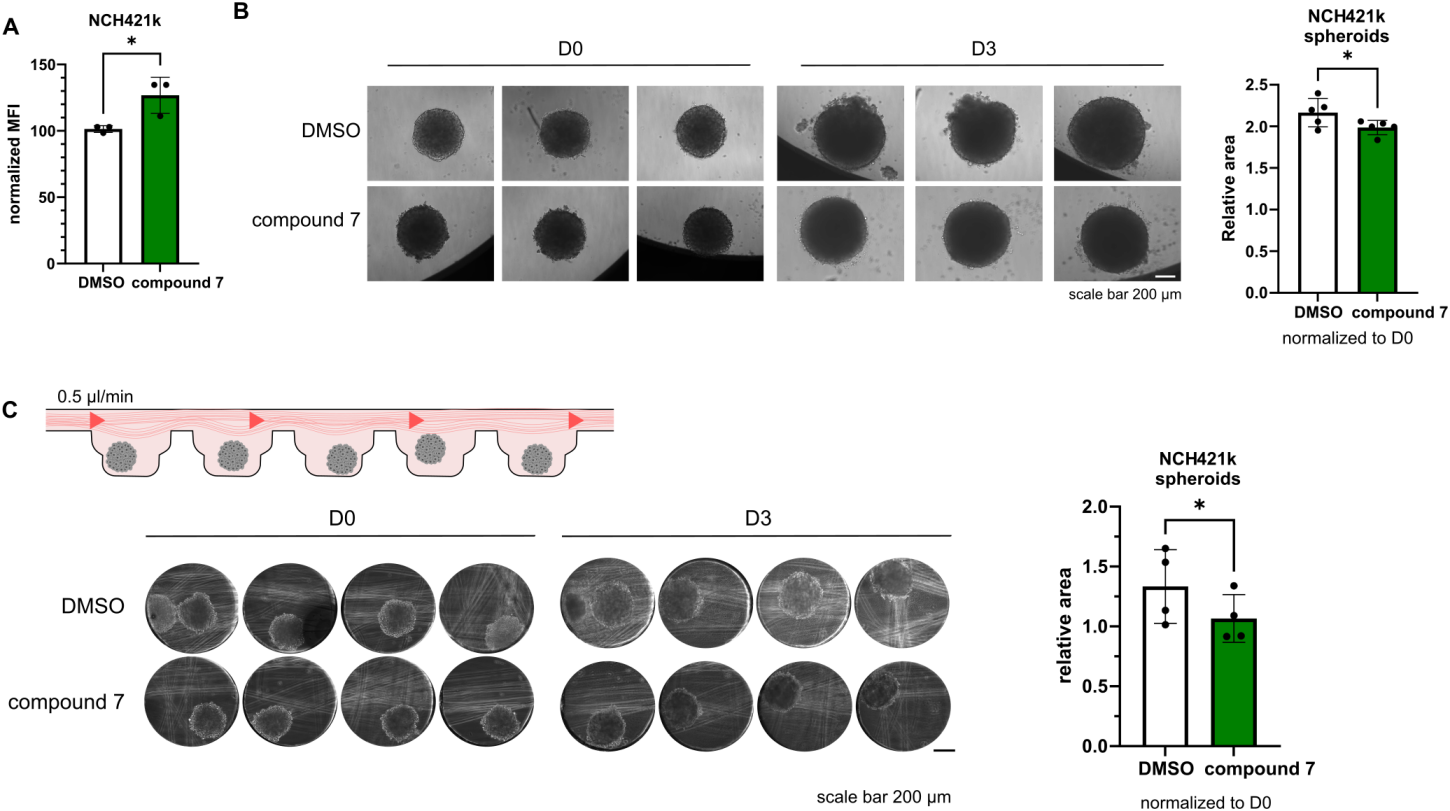
Effects of compound 7 on cancer cell proliferation. **(A)** NCH-421k cells treated with compound 7 show reduced proliferation compared with DMSO controls, as measured by CFSE labeling and flow cytometry. Graph shows normalized CFSE median fluorescence intensity (n=3). **(B)** Representative images of NCH-421k spheroids treated with DMSO or 20 µM compound 7 before (D0) and after 3 days of treatment (D3) cultured in static conditions (left). Quantification of spheroid area, normalized to D0 (n=5) (right) **C**: Schematic representation of the experiment (top left). Representative images of NCH-421k spheroids inside spheroid perfusion microfluidic chip, scale bar 200 µm (bottom left). Quantification of spheroid area, normalized to D0 (n=4) (right). *p<0.05.

Cathepsins activity can be related to both immune cells or cancer cells. Here we show that cathepsin V is expressed also by NCH-421k cells (**Supplementary Figure S4A**). In our experiments we have observed, that treatment of NCH-421k cells with compound 7 affects their proliferation in a standard flow cytometry CFSE proliferation assay (**Figure 4A**), in 3D static conditions (**Figure 4B**), and perfused conditions, when NCH-421k spheroids were incorporated inside the microfluidic device (**Figure 4C**), as measured by decreased spheroid surface area in treated condition compared to DMSO, control condition.

## 4 Discussion

In this study, we have demonstrated that cystatin F is a contributor to the immune suppression in GBM and plays a part in the functional impairment of immune cells. Our findings show that cystatin F expression correlates with immunosuppressive immune gene signatures in GBM, and that targeting its activation through inhibition of cathepsin V improves NK cell cytotoxic potential in 2D, 3D, and perfused *in vitro* models.

Analysis of gene expression data from the TIMER database revealed co-expression of *CST7* (encoding cystatin F) predominantly with immune-related genes. Clustering based on correlation strength identified three major gene groups: Cluster 1 includes genes with weak correlation with *CST7*. It contains tumor cell associated genes (*SOX2*, a marker of tumor stem cells, *GFAP* a marker of astrocytes and differentiated tumor cells, *CTSL2* a marker expressed in both tumor and immune cells), and some genes associated with immune activation (*KLRK1*, coding for NKG2D activating receptor on NK cells, *IL12A*, promoting Th1 differentiation, *LAMP1*, involved in the degranulation of cytotoxic immune cells, *NOS2*, which promotes inflammation). We have previously shown that cystatin F is expressed on the protein level in GBM tissue in various cell types, besides microglia/macrophages, also in tumor stem cells and differentiated tumor cells (16). From the negative correlation of *CST7* expression with tumor-associated genes, we may conclude that the increased cystatin F protein level in tumor cells was primarily due to the *in trans* activity of cystatin F. We have shown that both tumor and immune cells can internalize and retain active cystatin F from the microenvironment (15, 16, 38). Additionally, intratumoral heterogeneity with different GBM cell states could lead to protein expression in subsets of tumor cells not reflected in the bulk RNA profiles. Alternatively, other post-transcriptional modifications in tumor cells could affect *CST7* mRNA levels, despite higher protein content.

As tumor samples are heterogeneous, it is important to consider the correlations between immune genes also in terms of tumor composition, in order to eliminate correlations to appear simply because of the increased immune infiltration in the samples. Cluster 2 contains genes with moderate correlation with *CST7*, and contains genes with partially functional immune responses (such as NK cell activation receptors (*NCR1, NCR3, CD226*), inhibitory receptors (*KLRC1* and *KIR2DL1*), cytokine genes (*IFNG, TNF*), and genes connected with cell adhesion (*CD44*). Cluster 3 contains genes with strong *CST7* correlations. Genes include general microglia/macrophages genes (*AIF1, CD68, ITGAM*) and M2-associated gene signatures (*CD163, CSF1R, TREM2, MRC1* (CD206)), suggesting immunosuppressive myeloid infiltration. Additionally, cluster 3 contains activation markers (*GZMB, PRF1, CD69* and *CD86*), but functionally impaired cytotoxic immune cell responses, with immune checkpoint genes (*HAVCR2*, *PDCD1* and *TIGIT*). A strong correlation with *CST7* is maintained after adjusting for tumor purity (*PDCD1, TIGIT*). We can conclude that *CST7* expression is more closely linked to the immunosuppressive rather than activated immune environment in GBM. Furthermore, we have confirmed the co-expression of cystatin F with immune-related markers in GBM patients’ tissue sections, such as CD45, LAG3, TIM3, PD1, and DNAM1. The infiltration of immune cells in the selected tissue samples was limited and restricted to the areas surrounding the vessels. We have also observed reduced functionality in terms of cytotoxic function and cytokine secretion in NK cells isolated from GBM patients compared to healthy donors. While this was already confirmed for other cancer types, it is important when considering NK cells as a potential anti-cancer therapy for GBM patients. As NK cells rely on the balance between the interactions of activation and inhibitory surface receptors with their ligands on target cells, instead of antigen pre-sensitization, they are less likely to cause graft vs. host disease (52). Application of fully functional NK cells derived from healthy donors is advantageous over autologous NK cell therapy, and provides a possibility for off-the-shelf use, improving accessibility (53).

Due to N-glycosylation, cystatin F can be secreted from one cell type and internalized into another, where it can inhibit cysteine cathepsins (15, 17). Microglia and macrophages are a predominant immune population in GBM (54), and cystatin F expression in GBM has already been associated with this immune type (55, 56). In GBM, microglia and infiltrating macrophages interact with NK cells to shape the immune landscape, and targeting this crosstalk may shift the TME toward a more effective anti-tumor immune response (57, 58). By modifying the HMC3 microglial cell line to express cystatin F, we were able to obtain a relevant source of cystatin F in the cell model of TME. In co-culture experiments, we were able to show that cystatin F negatively impacts the cytotoxicity of primary NK cells, which were consequently less successful in eliminating the GSC cell line NCH-421k. In this model, we were able to better recapitulate the interplay between different cellular types regarding cystatin F release compared to models using conditioned media or the addition of recombinant proteins, which we have used in our previous studies.

Regardless of its cellular source, the presence of cystatin F can negatively impact the activity of cytotoxic immune cells. Using a selective, reversible, non-covalent inhibitor of cathepsin V (compound 7), we can effectively reduce the activation of cystatin F. Using this approach, we were able to improve NK cell cytotoxic function in standard NK cell cytotoxicity assays, as well as in more complex tumor models, such as static and perfusion GSC spheroids models in microfluidic chips. We have used two different dyes to measure the efficacy of NK cell killing in our 3D assays. In the static model we have used CFSE labeled spheroids and applied the reduction of CFSE intensity as a measure of cell death. As CFSE intensity is also affected by cell proliferation rate, we have tried to mitigate this confounding factor by treating spheroids with either DMSO or compound 7 as control condition. In order to remove this ambiguity, we have introduced a cell death specific dye; Cytotox Green, in our perfused model.

Interestingly, we could also observe an effect on IFNγ secretion, which was decreased after treatment with compound 7. Unlike cathepsin L or cathepsin S, cathepsin V is less studied in immune cells and not directly involved in classical antigen processing for MHC class II presentation. There is no evidence that cathepsin V is directly involved in IFNγ synthesis or secretion, but it may have indirect regulatory roles in immune cell function that could influence cytokine production. Similarly, there is no experimental evidence of direct involvement of cystatin F in the regulation of IFNγ secretion. The currently available studies only implicate crosstalk and indirect effects on IFNγ via altered activation states and cell-cell interactions. For example, cystatin F was found to be upregulated in inflamed/viral contexts in neutrophils (59), and treatment with inflammatory cytokines, including IFNγ, decreased cathepsin V expression in human bronchial epithelial cells (60). Focusing on NK cells in antitumor immune response, the cytotoxic function and cytokine release function are changing at different rates. From fully functional state with intact cytotoxic and cytokine release abilities, NK cells first lose their cytotoxic ability while retaining (or even increasing) their ability to secrete IFNγ- a state referred to as split-anergy. With further interactions with the TME NK cells become dysfunctional (61). We have shown in our previous studies, that in the stage of split anergy, cystatin F levels are upregulated (9). Similarly, primary NK cells treated with recombinant CSTF had increased IFNγ secretion (17). According to our previous studies, active cystatin F therefore skews NK cells towards a low cytotoxic state, but with a higher IFNγ release capacity. In this study, we have observed an inverse effect, where less active cystatin F, caused by cathepsin V inhibition, shifts NK cells toward a cytotoxic, but lower IFNγ releasing state. However, again, we could not find a direct regulatory link between cystatin F and IFNγ regulation. Transcriptional regulation was shown to be a role for some cysteine cathepsins, however, whether cathepsin V influences cytokine gene expression by modifying signaling molecules remains to be elucidated.

Our work was focused mainly on the involvement of cathepsin V in cystatin F activation in NK cells; however, we have observed that compound 7 also impaired the proliferation of GSCs, which is in agreement with studies implicating cathepsin V in the regulation of cell proliferation (21, 22). Knockdown of CTSL2 blocked the cell cycle and DNA replication in hepatocellular carcinoma, and delayed progression through the G2/M phase in breast cancer cell lines (23, 27). Furthermore, CTSL2 expression correlated with several cell cycle and growth regulatory genes (Ki-67, cyclin B1, HER2 receptor tyrosine kinase) and controls the nuclear expression levels of H3 and H4 histones (22, 23). However, at this time, the exact mechanism through which cathepsin V promotes cell proliferation in NCH-421k cells remains to be elucidated.

We aimed to evaluate our NK cell modulation strategy using different *in vitro* models. Besides 2D cell co-cultures we used static and perfused 3D spheroid cultures to provide a platform more relevant to *in vivo* tumor environment. Using microfluidic chips, it is important to consider several aspects, whether to mimic several compartments (tumor, blood vessels…), whether we want the cells to be exposed to shear stress or interstitial flow/diffusion, how many cell types we wish to incorporate, and if we want to add extracellular matrix components. Here we have used relatively simple models of microfluidic chips with multiple compartments, which allowed us to simulate vessels and tumor tissue, allowing us to mimic the infiltration/migration of NK cells to the tumor. Since solid tumors experience interstitial flow (62, 63), rather than shear stress, which is more relevant in the vasculature (64), we selected microfluidic chips that replicate these conditions. We used either chips, which generate interstitial flow (51), or have such an architecture that enables diffusion of nutrients to the spheroids, without exposing them to shear stress. Our models may at least partially substitute mouse models as they usually lack relevant immune cells (65, 66). There are also considerable differences between the mouse and human immune system (67), and importantly, cathepsin V is not expressed in mice. This represents a major limitation for conventional *in vivo* validation, as there are no direct orthologs of cathepsin V in mice (68). Therefore, only the use of highly advanced humanized mouse models would enable us to study the effects of cathepsin V inhibitor in *in vivo* setting (69, 70). However, humanized mice models are technically demanding. In this context the use of advanced *in vitro* models using patient derived material such as organoids, precision cut tumor slices, tumor fragments and primary immune cells in innovative *in vitro* settings using perfused microfluidic platforms allows us to overcome species specific limitations and enables testing of therapeutic strategies in an intermediate level of physiological relevance, providing a more relevant and selective process for selecting a best strategy for potential therapy. Recent changes in FDA regulations also support the use of advanced *in vitro* models in order to reduce animal use, providing a bridge between early discovery and preclinical translation (71, 72). Nonetheless, moving towards potential clinical use of cathepsin V targeting will require integration of data from both *in vitro* and *in vivo* studies in order to validate efficacy, safety and specificity, which may increase the preclinical development timeline before moving to clinical research.

To better recapitulate the GBM TME, we have to further improve our models to include several cellular types in addition to GSCs. This would enable us to study possible interactions with other cell types and possible off-target effects. By culturing our models for a longer time, we would also be able to observe the longevity of the observed effects.

In conclusion, our findings identify cystatin F as an immunosuppressive agent in the GBM microenvironment. By targeting cathepsin V and therefore reducing cystatin F activation in NK cells, we can improve their cytotoxic function against GSCs, which we have confirmed using standard assays, as well as in 3D models and perfused models. These results highlight the relevance of cystatin F as a regulator of immune suppression in GBM and point towards cathepsin V as a potential therapeutic target. In future studies, incorporating multiple cellular components into our models will enable a more comprehensive evaluation of cystatin F mediated immune suppression and potential therapy interventions.

## Data Availability

The raw data supporting the conclusions of this article will be made available by the authors, without undue reservation.
