# Supplementary material for: Targeting cystatin F activation enhances NK cell cytotoxicity in glioblastoma models
Source: Front Immunol. 2025 Oct 28;16:1708281. doi: 10.3389/fimmu.2025.1708281 (PMC12602517; doi:10.3389/fimmu.2025.1708281)
Supplement: Supplementary file 1 [file Table1.docx]

**Supplementary Material**

**Targeting Cystatin F Activation Enhances NK Cell Cytotoxicity in Glioblastoma Models**

**Emanuela Senjor^1,2^, Anamarija Habič^3,4^, Urban Švajger^5^, Ana Mitrović^1,2^, Matic Proj^2^, Andrej Porčnik^6^, Borut Prestor^6^, Miha Jerala^7^, Matic Bošnjak^7^, Stanislav Gobec^2^, Barbara Breznik^3,8^, Janko Kos^1,2^, Milica Perišić Nanut^1*^**

^1^ Jožef Stefan Institute, Department of Biotechnology

^2^ Faculty of Pharmacy, University of Ljubljana

^3^ National Institute of Biology, Department of Genetic Toxicology and Cancer Biology, Ljubljana, Slovenia

^4^ Jožef Stefan International Postgraduate School, Ljubljana, Slovenia

^5^ Slovenian Institute for Transfusion Medicine, Ljubljana, Slovenia

^6^ Department of Neurosurgery, University Medical Centre Ljubljana, Ljubljana, Slovenia

^7^ Institute of Pathology, Faculty of Medicine, University of Ljubljana, Ljubljana, Slovenia

^8^ Faculty of Chemistry and Chemical Engineering, University of Ljubljana

| **Supplementary Figures**  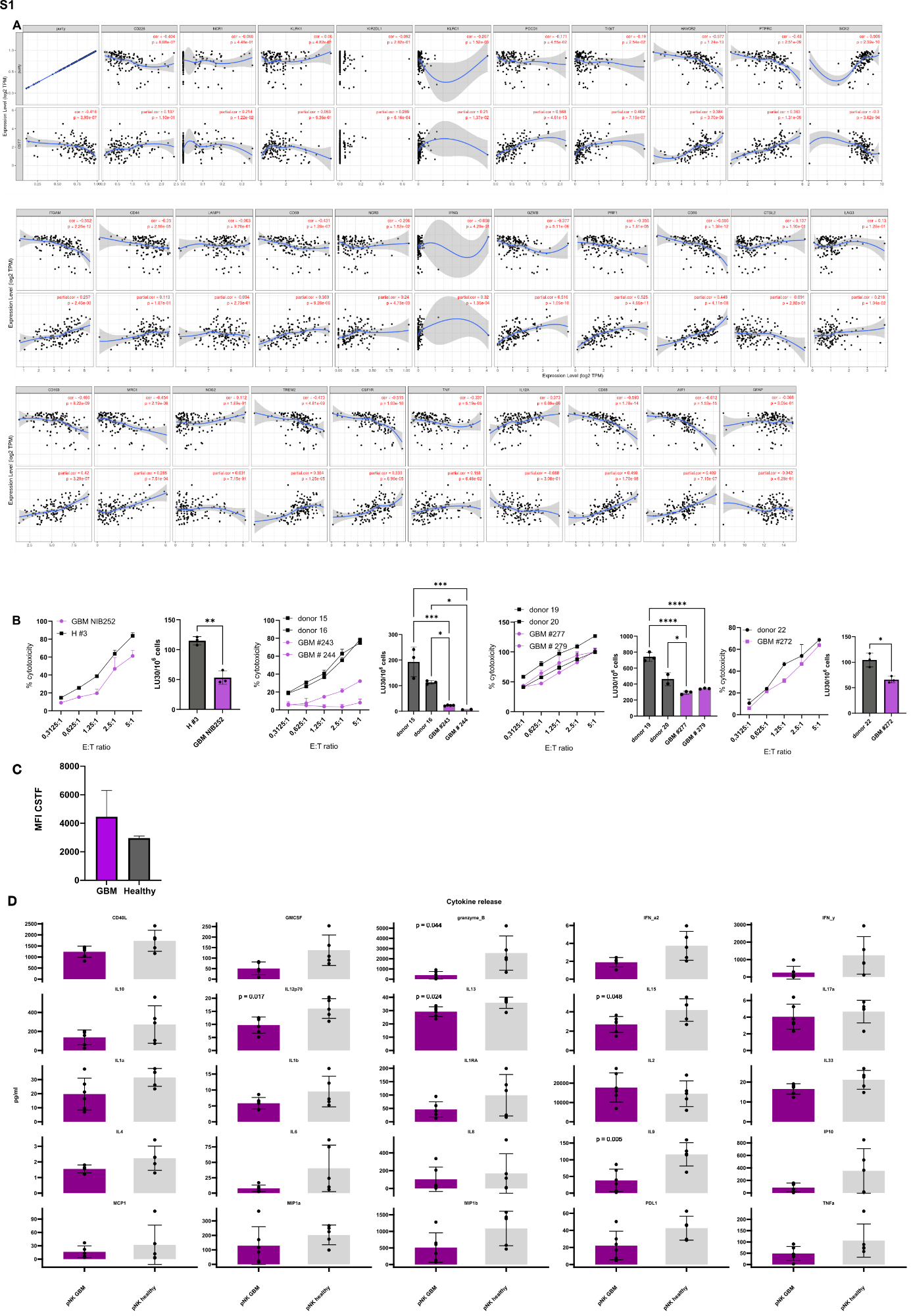 |
| --- |
| **Supplement Figure 1**  **A**: TIMER database results of correlation of various immune genes with tumor purity and cystatin F in glioblastoma. **B**: % cytotoxicity plots for calcein release cytotoxicity assay of patients’ and healthy donors NK cells. **C:** Flow cytometry analysis of cystatin F expression in primary NK cells isolated from glioblastoma patients or healthy donors (n=2). **D:** Detailed representation of cytokine secretion of primary NK cells from glioblastoma patients (purple bars) and healthy donors (grey bars). P values are provided where the differences are significant. * p<0.05. |

| 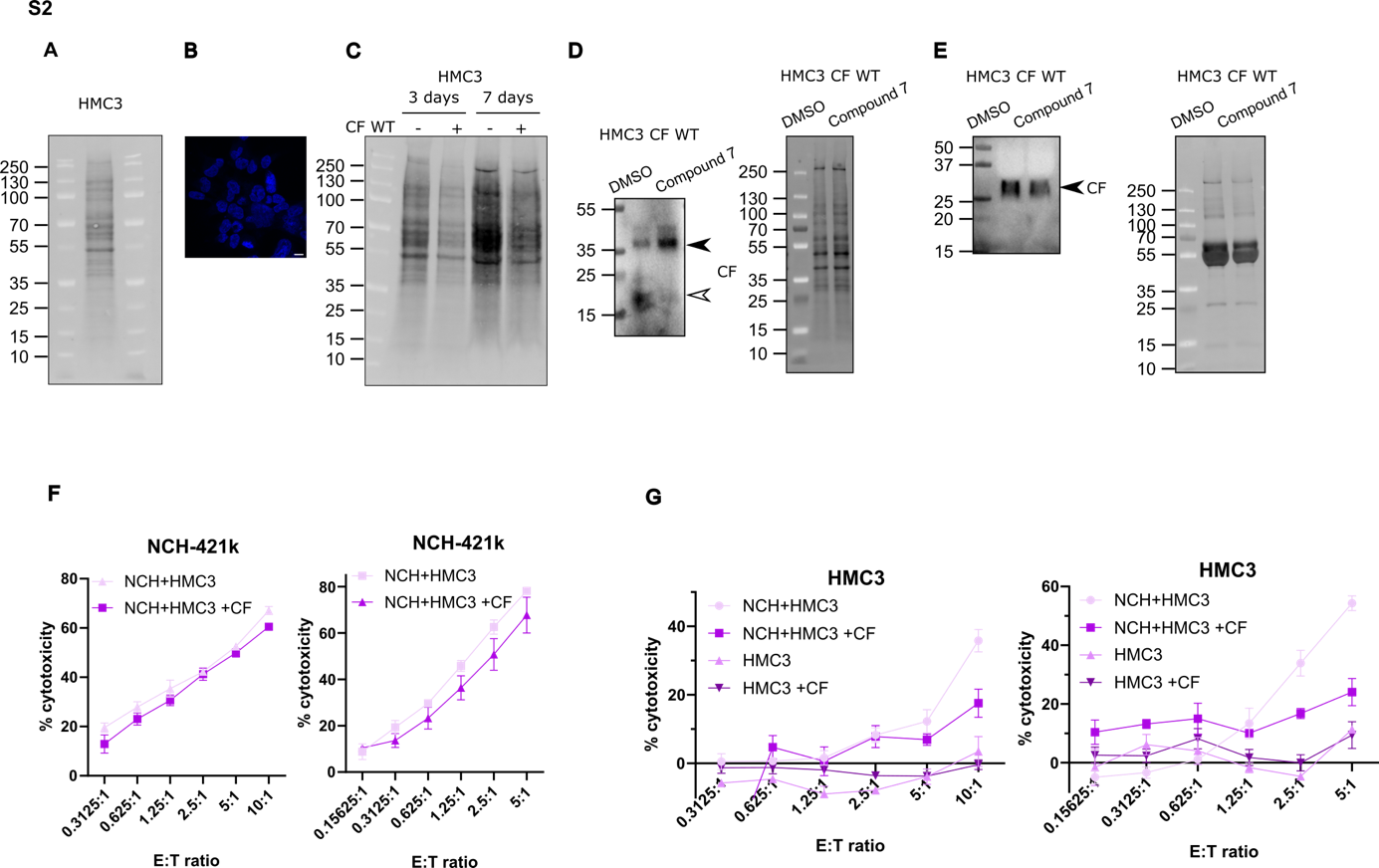 |
| --- |
| **Supplement Figure 2**  **A**: Stain free blot of protein loading for western blot in Figure 2A. **B**: Secondary antibody control for HMC3 staining of cathepsin V. Scale bar 10 µm. **C**: Stain free blot of protein loading for western blot in Figure 2C. **D:** In transfected HMC3 CF WT cells treated with compound 7 the conversion of cystatin F to monomeric form in HMC3 cell line is limited (left). Stain free loading control (right). **E:** Transfected HMC3 cell line also secretes cystatin F in the cell media (left). Stain free loading control (right). **F:** % cytotoxicity plots for flow cytometry cytotoxicity experiment, that were used for the calculation of LU30 for NCH-421k cells. **G:** % cytotoxicity plots for flow cytometry cytotoxicity experiment, showing HMC3 cells are poor targets for NK cell cytotoxicity.* p<0.05. |

| 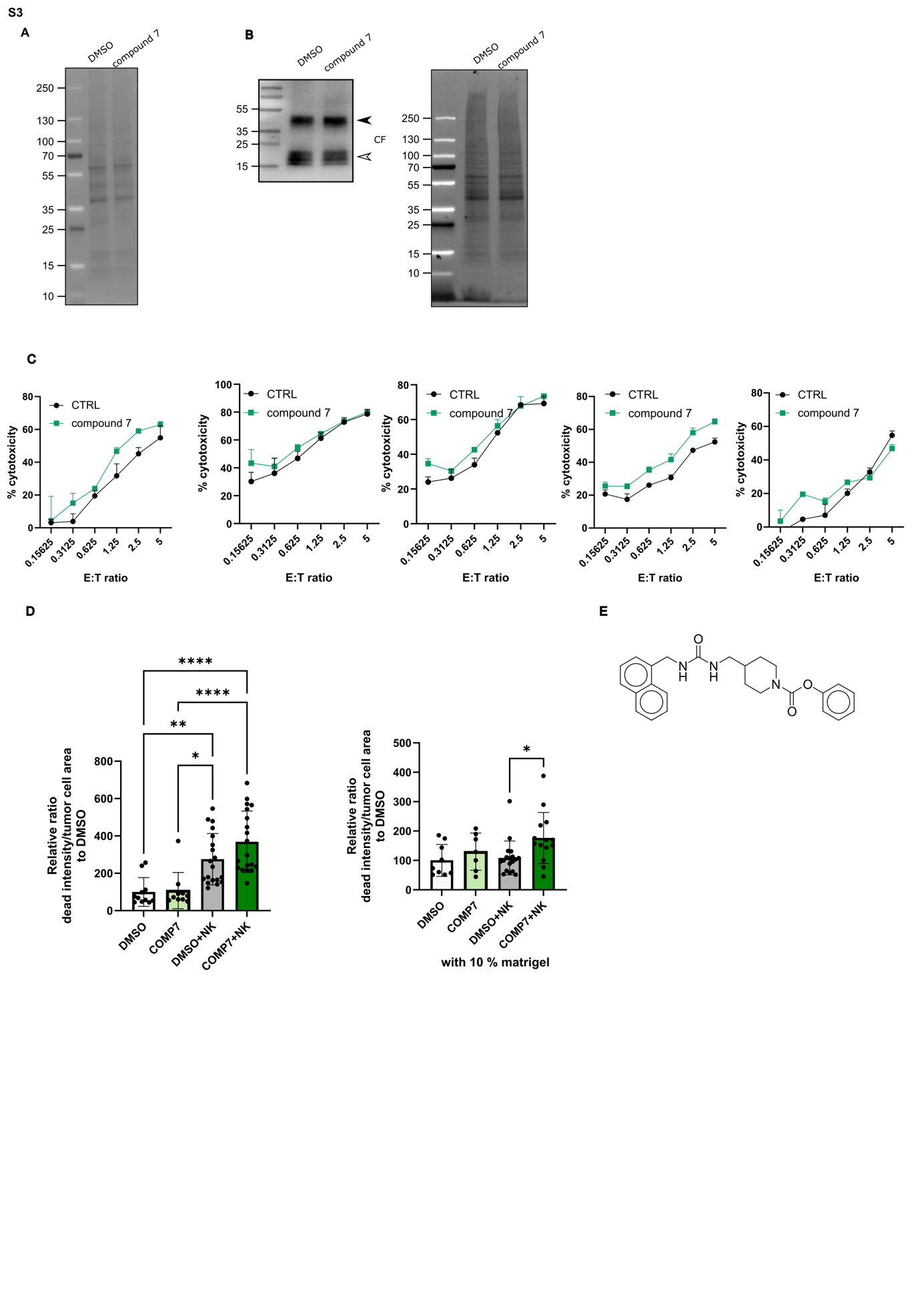 |
| --- |
| **Supplement Figure 3**  **A**: Stain free blot showing protein loading for the blot presented in Fig 3A. **B**: Additional western blot (left) and stain free blot of protein loading (right) for other donors’ pNK treated with compound 7. Arrowheads point to the dimeric form (black) and monomeric form (white). **C**: % cytotoxicity plots for pNK flow cytometry cytotoxicity experiment, that were used for the calculation of LU30. D: Additional individual experiments of cytotoxicity experiments of NCH-421k cells treated with pNK cells and compound 7 (20 µM, 18h) for culture conditions without extracellular matrix and the addition of 10% matrigel to the microfluidic device (right). Each dot represents a field of view. * p<0.05. E: Structure of compound 7. |

| **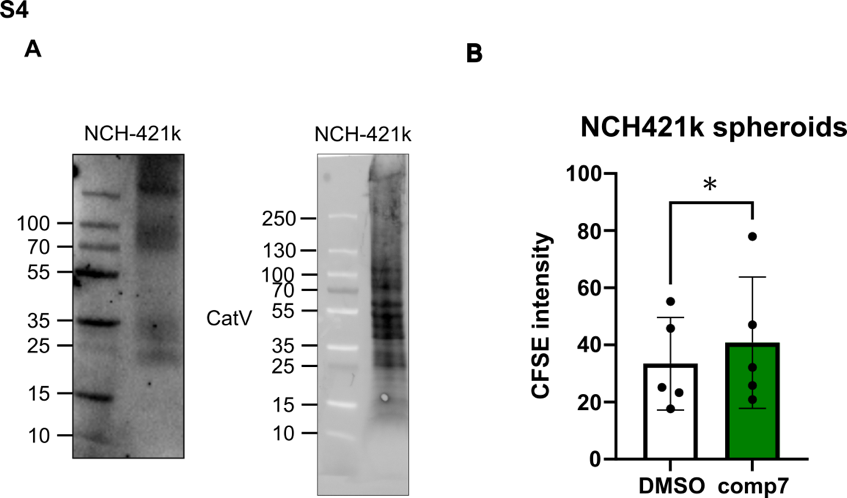** |
| --- |
| **Figure S4: Cytotoxic potential of NK cells can be improved by preventing the activation of cystatin F.**  A: NCH-421k cells expression of cathepsin V (left). Stain free loading control (right). CFSE intensity of NCH-421k spheroids treated with DMSO or compound 7 in static conditions at day 3. * p<0.05. |
